# Representation of Perceptual Evidence in the Human Brain Assessed by Fast, Within-Trial Dynamic Stimuli

**DOI:** 10.3389/fnhum.2020.00009

**Published:** 2020-02-04

**Authors:** Sebastian Bitzer, Hame Park, Burkhard Maess, Katharina von Kriegstein, Stefan J. Kiebel

**Affiliations:** ^1^Department of Psychology, Technische Universität Dresden, Dresden, Germany; ^2^Department for Cognitive Neuroscience, Faculty of Biology, Bielefeld University, Bielefeld, Germany; ^3^Center of Excellence Cognitive Interaction Technology, Bielefeld University, Bielefeld, Germany; ^4^Brain Networks Group, Max Planck Institute for Human Cognitive and Brain Sciences, Leipzig, Germany; ^5^Max Planck Research Group Neural Mechanisms of Human Communication, Max Planck Institute for Human Cognitive and Brain Sciences, Leipzig, Germany

**Keywords:** MEG (magnetoencephalography), posterior cingulate cortex (PCC), perceptual decision making, decision evidence, event-related regression, within-trial fluctuations

## Abstract

In perceptual decision making the brain extracts and accumulates decision evidence from a stimulus over time and eventually makes a decision based on the accumulated evidence. Several characteristics of this process have been observed in human electrophysiological experiments, especially an average build-up of motor-related signals supposedly reflecting accumulated evidence, when averaged across trials. Another recently established approach to investigate the representation of decision evidence in brain signals is to correlate the within-trial fluctuations of decision evidence with the measured signals. We here report results of this approach for a two-alternative forced choice reaction time experiment measured using magnetoencephalography (MEG) recordings. Our results show: (1) that decision evidence is most strongly represented in the MEG signals in three consecutive phases and (2) that posterior cingulate cortex is involved most consistently, among all brain areas, in all three of the identified phases. As most previous work on perceptual decision making in the brain has focused on parietal and motor areas, our findings therefore suggest that the role of the posterior cingulate cortex in perceptual decision making may be currently underestimated.

## 1. Introduction

During perceptual decision making observers make inference about the state of their environment. Supported by findings in single neurons of non-human primates, the underlying mechanism has been characterized as an accumulation-to-bound process (Gold and Shadlen, 2007). Specifically, the current consensus is that during perceptual decision making the brain accumulates noisy pieces of sensory evidence across time until it reaches a confidence bound. Most experimental results on this process have been based on stimuli which have been designed to provide the same amount of evidence per unit time on average across trials. Trial-averaged accumulated evidence then should follow a gradual build-up with evidence-dependent slope and a maximum close to the response within trial (Gold and Shadlen, 2007).

In humans, evidence of this kind of average build-up have been found using magneto- and electroencephalography (M/EEG). For example, lateralized oscillatory signals in the beta band measured with magnetoencephalography exhibit this build-up, where sources were located to dorsal premotor and primary motor cortex (Donner et al., 2009). In EEG, there are similar findings of a build-up for lateralized readiness potentials and oscillations (de Lange et al., 2013; Kelly and O'Connell, 2013). Furthermore, when human participants have to detect the presence of stimuli in noise, a centro-parietal positivity shows the characteristics of an evidence-dependent build-up independently of the type of stimulus used and the kind of response made (O'Connell et al., 2012; Kelly and O'Connell, 2013). Together these findings suggest that the human parietal and motor cortices are involved in perceptual decision making and in particular represent accumulated evidence. This view is compatible with electrophysiological recordings in non-human animals (Hanks and Summerfield, 2017) and with an active role of the motor system during decision making (Cisek and Kalaska, 2010).

It has long been known that electromagnetic signals over motor areas build up toward a motor response and can signal an eventual choice even before the response (Smulders and Miller, 2012). This means that the crucial aspect of decision evidence representations is not the build-up as such, but its covariation with the theoretically available evidence. Consequently, more recent approaches have induced consistent, within-trial changes in available decision evidence (Wyart et al., 2012; Brunton et al., 2013; Thura and Cisek, 2014; Hanks and Summerfield, 2017). These within-trial changes allow for more specific analyses, because one can directly assess the covariation between decision evidence and neural signals (a) across a much richer sample of evidences than available with the trial-constant evidences in previous analyses and (b) while the decision is ongoing.

Although it has previously been shown that electromagnetic signals in the human brain correlate with within-trial changing decision evidence (de Lange et al., 2010; Gould et al., 2012; Wyart et al., 2012; Gluth et al., 2013), these studies had either rather long stimulus presentation times atypical for fast perceptual decisions (Gould et al., 2012; Gluth et al., 2013), or did not employ a reaction time paradigm (de Lange et al., 2010; Gould et al., 2012; Wyart et al., 2012). In the present work we therefore sought representations of decision evidence in a two-alternative forced choice reaction time paradigm in which we induced changes in decision evidence every 100 ms. That is, our paradigm aims at mimicking natural perceptual decision making behavior more closely than previous investigations with controlled, within-trial changing evidence while still observing neural responses across the whole human brain.

Specifically, we investigated correlations between decision evidence and human MEG signals and their sources. We found particularly large effects of decision evidence in the human MEG in three consecutive phases after a particular piece of evidence became available. The underlying sources indicate that the information delivered by the evidence propagated, as expected, from visual over parietal to motor areas. In addition, our results implicate posterior cingulate cortex in all of three identified phases suggesting a central role of this brain region in the transformation of sensory signals to decision evidence in our task.

## 2. Materials and Methods

### 2.1. Experimental Design

#### 2.1.1. Participants

Thirty-seven healthy, right-handed participants were recruited from the Max Planck Institute for Human Cognitive and Brain Sciences (Leipzig, Germany) participant pool (age range: 20–35 years, mean 25.7 years, 19 females). All had normal or corrected-to-normal vision, and reported no history of neurologic or psychiatric disorders. One participant was excluded from MEG measurement due to low performance during training. In total, 36 participants participated in the MEG study. Two participants' data were excluded from analyses due to excessive eye artifacts and too many bad channels. Finally, 34 participants' data were analyzed (age range: 20–35 years, mean 25.85 years, 17 females).

#### 2.1.2. Stimuli

In each trial, a sequence of up to 25 white dots were presented on a black screen. Each dot was displayed for 100 ms (6 frames, refresh rate 60 Hz). The white dots were located at x, y coordinates which were sampled from one of two-dimensional Gaussian distributions with means located at ±25 pixels horizontal distance from the center of the screen. The standard deviation was 70 pixels in both axes of the screen. The mean locations were the two target locations [(–25, 0): left, (25, 0): right]. These target locations corresponded to visual angles ±0.6° from the center of the screen. The standard deviation of the Gaussian distribution corresponded to ±1.7°. See Figure 2A for a visualization of the stimulus.

We reused a subset of stimuli generated for a previous behavioral study (Park et al., 2016). From this study we know that some white dot sequences (also called “stimuli,” or “trials”) tend to lead to long response times (RTs) across participants while others lead to short RTs, as expected. To increase the statistical power of within-participant analyses in the present study, i.e., to increase the amount of MEG data available while participants observe the stimulus, we selected 28 trials (from a total of 200 trials) from the previous study which tended to have the longest RTs (~70% of the participants had RTs > 700 ms). These stimuli were copied 6 times and modified, as described below, to result in a set of 168 stimuli/trials. We further added 72 stimuli which had the lowest average RTs across subjects in the previous study as catch trials, to prevent participants from adapting to the long RT stimuli. We then duplicated this resulting set of 240 stimuli by mirroring the x-coordinate of each white dot position, e.g., *x* = 5 became *x* = −5. This manipulation ensured that irrespective of the choice tendencies for the selected individual stimuli, e.g., participants may have tended to respond “left” for a stimulus, the stimulus set was balanced across responses and had no behavioral bias.

When designing the study, we further aimed to ensure that the 28 long RT stimuli contained a large, regular variation of x-coordinates roughly half-way through the trial. This was supposed to lead to corresponding variation in MEG signals that could be easily picked up by an across-trial regression analysis. We implemented this by manipulating the x-coordinate of the 5th dot in each of the long RT white dot sequences. Especially, from each one of these sequences we created 6 variants in which the 5th dot x-coordinate took on values –160, –96, –32, 32, 96, and 160 (pixels) while all other dot positions were unaltered so that any unexpected, stimulus-specific effects were equal across the 6 variants. To prevent that participants notice that the 5th dot took on only one of 6 possible x-coordinates in roughly two thirds of the trials, we pseudo-randomized the trial order for each participant so that long RT stimuli were randomly interleaved with short RT stimuli. In a post-experiment questionnaire no participant reported to have noticed any regularity in the stimuli.

In a preliminary analysis we found that the natural variation of the stimuli, e.g., of the first dot, already induced observable effects in the MEG. To increase statistical power, we, consequently, included all trials and dot locations for analysis, as described below, and did not analyse the 5th dot in any special way.

#### 2.1.3. Procedure

Participants were seated in a dimly lit shielding room during the training and the MEG measurement. Visual stimuli were presented using Presentation® software (Version 16.0, Neurobehavioral Systems, Inc., Berkeley, CA, www.neurobs.com). The display was a semi-transparent screen onto which the stimuli were back-projected from a projector located outside of the magnetic shielding room (Vacuumschmelze Hanau, Germany). The display was located 90 cm from the participants. The task was to find out which target (left or right) was the center of the white dot positions. Participants were instructed as follows: Each target represented a bee hive and the white dot represented a bee. Participants should tell which bee hive is more likely the home of the bee. They were additionally instructed to be both accurate and fast, but not too fast at the expense of being inaccurate, and not too slow that the trial times out. Participants went through a minimum 210 and maximum 450 trials of training, until they reached a minimum of 75% accuracy. Feedback (correct, incorrect, too slow, too fast) was provided during the training. After training, a pseudo-main block with 200 trials without feedback preceded MEG measurement. After the pseudo-main session, the 480 trials in randomized order were presented to each participant divided into 5 blocks. The MEG measurement lasted ca. ~ 60 min, including breaks between blocks. Each trial started with a fixation cross (randomized, 1,200–1,500 ms uniform distribution) followed by two yellow target dots. After 700 ms, the fixation cross disappeared and the first white dot appeared. The white dot jumped around the screen and stayed at each location for 100 ms, until the participant submitted a response by pressing a button using either hand, corresponding to the left/right target, or when the trial timed-out (2.5 s). See Figure 2A for a visualization of the sequence of events in a trial. In order to maintain motivation and attention throughout the measurement, participants were told to accumulate points (not shown to the participants) for correct trials and adequate (not too slow and not too fast, non-time-out) RTs. Bonus money in addition to compensation for participating in the experiment were given to participants with good performances. RTs and choices were collected for each trial for each participant. Although the trial order was randomized across participants, every participant saw exactly the same 480 trials.

### 2.2. Definition of Momentary and Accumulated Evidence

Momentary and accumulated decision evidence are theoretical constructs defined through a model of the decision process applied to the specific task at hand. For our task it can be shown that momentary evidence corresponds to the signed x-coordinate values of the white dot positions and accumulated evidence can be equated with the simple cumulative sum of these x-coordinates across the sequence of dot positions. The model used to define this is an ideal observer model for this task that we described in earlier publications (Bitzer et al., 2014; Park et al., 2016).

It may be surprising that the y-coordinates and the two targets do not directly contribute to decision evidence. This results from the symmetries of the task: Both targets are on the same horizontal line meaning that only the x-coordinate separates the two targets and thus is sufficient to decide between the targets. Because the targets are mirrored across the y-axis, i.e., have the same distance to the screen center, any white dot on the left side of the screen is evidence for the left target and any white dot on the right side is evidence for the right target -in proportion to the distance to the screen center. This means that the raw x-coordinate values are valid measures of decision evidence without explicit reference to the targets.

### 2.3. MEG Data Acquisition and Preprocessing

MEG data were recorded with a 306 channel Vectorview™ device (Elekta Oy, Helsinki, Finland), sampled at 1,000 Hz. The MEG sensors covered the whole head, with triplet sensors consisting of two orthogonal gradiometers and one magnetometer at 102 locations. Additionally, three electrode pairs were used to monitor eye movement and heart beats at the same sampling rate. The raw MEG data was corrected for head movements and external interferences by the Signal Space Separation (SSS) method (Taulu et al., 2005) implemented in the MaxFilter™ software (Elekta Oy) for each block. The subsequent preprocessing was performed using MATLAB (Mathworks, Massachusetts, United States). The head movement corrected data was high-pass and low-pass filtered using a linear phase FIR Kaiser filter (corrected for the shift) at cut-off frequencies of 0.33 and 45 Hz respectively, with filter orders of 3,736 and 392, respectively. The filtered data was then down-sampled to 250 Hz. Then independent component analysis (ICA) was applied to the continuous data using functions in the EEGLAB (Delorme and Makeig, 2004) to remove eye and heart beat artifacts. The data dimensionality was reduced by principal component analysis (PCA) to 50 or 60 components prior to running the ICA. Components which had high temporal correlations (>0.3) or typical topographies with/of the EOG and ECG signals were identified and excluded. On average 4.6 components (SD: 2.8) were removed (excluding data from 2 participants, which were not saved) during this initial ICA. The ICA-reconstructed data for each block was combined, and epoched from –300 to 2,500 ms from the first dot onset (zero). Another ICA was applied to these epoched data in order to check for additional artifacts and confirm typical neural topographies from the components. The data dimensionality was reduced by PCA to 40 components, and on average 2.1 components (SD: 2.0) were removed across all participants. The ICA reconstructed data and original data were compared and inspected in order to ensure only artifactual trials were excluded. Before statistical analysis we used MNE-Python v0.15.2 (Gramfort et al., 2013, 2014) to downsample the data to 100 Hz (10 ms steps) and perform baseline correction for each trial where the baseline value was the mean signal in the period from –300 to 0 ms (first dot onset).

### 2.4. Source Reconstruction

We reconstructed the source currents underlying the measured MEG signals using noise-normalized minimum norm estimation (Dale et al., 2000) implemented in the MNE software. We conducted source reconstruction on single trial measurements, not on evoked signals. To create participant-specific forward models we semi-automatically co-registered the head positions of participants with the MEG coordinate frame while at the same time morphing the participants' head shape to that of Freesurfer's fsaverage by aligning the fsaverage head surface to a set of head points recorded for each participant. We defined a source space along the white matter surface of the average subject with 4098 equally spaced sources per hemisphere and an approximate source spacing of about 5 mm (MNE's "oct6" option). For minimum norm estimation we assumed a signal-to-noise ratio of 3 (lambda2 = 0.11). We estimated the noise covariance matrix for noise normalization (Dale et al., 2000) from the MEG signals in the baseline period spanning from 300 ms before to first dot onset in each trial. We further used standard loose orientation constraints (loose = 0.2), but subsequently picked only the currents normal to the cortical mantle. We employed standard depth weighting with a value of 0.8 to overcome the bias of minimum norm estimates toward superficial sources. We computed the inverse solution from all MEG sensors (magnetometers and the two sets of gradiometers) returning dynamic statistical parametric maps for each participant. Before some of the subsequent statistical analyses we averaged the reconstructed source signals across all sources of a brain area as defined by the HCP-MMP parcellation of the human connectome project (Glasser et al., 2016).

### 2.5. Statistical Analysis

#### 2.5.1. Regression Analyses

Most of our results were based on regression analyses with a general linear model giving event-related regression coefficients (Hauk et al., 2006; Clarke et al., 2013). We differentiate between (i) a standard regression analysis on events aligned at the time when the white dot appeared in each trial, (ii) expanded regression analyses on events aligned at the times of white dot position changes, and (iii) response-aligned regression analyses.

In sensor space we opted to apply regression analyses only to magnetometer measurements to avoid additional complexity in the models induced by trying to properly combine measurements from magnetometers and planar gradiometers. Solutions for this problem are already built into source reconstruction procedures so that regression analyses automatically used all sensor data when applied in source space.

##### 2.5.1.1. Standard regression analysis

In the standard regression analysis we defined dot-specific regressors with values changing only across trials. For example, we defined a regressor for momentary evidence (x-coordinate) of the 2nd white dot position presented in the trial. For brevity we also call white dot positions (1st, 2nd, and so forth in the sequence of dot positions) simply “dots.”

We only report results of a standard regression analysis in Figure 4. The data for this analysis were the preprocessed magnetometer time courses. This analysis included the dot x- and y-coordinates of the first 6 dots as regressors of interest (in total 12 regressors). Additional nuisance regressors were: the response of the participant, a participant-specific trial count roughly measuring time within the experiment and an intercept capturing average effects.

##### 2.5.1.2. Expanded regression analyses

**Figure 1:**
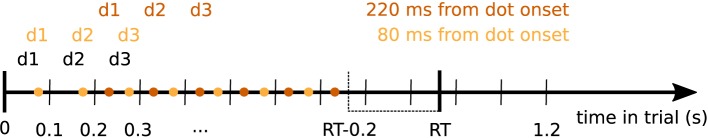
Diagram demonstrating the selection of data points entering the expanded regression analyses. Dot positions (d1, d2, d3, …) changed every 100 ms in the experiment (black). Colored dots indicate times at which signal data points entered the analysis for a given time from dot position change (dot onset, shown exemplarily for 80 and 220 ms from dot onset). We only considered time points up to 200 ms before the response in each trial. Colored d1, d2, d3 above the points indicate the dot positions associated with the corresponding signal data points for the given time from dot onset. For each trial, these pairs of signal data and dot positions entered the expanded regression analyses.

Expanded regression analyses were based on an expanded set of data created by dividing up the data into partially overlapping epochs centered on the times of dot position changes. For each time point after this dot onset the data contained a variable number of time points depending on how many more dots were presented in each individual trial before a response was given by the participant. For example, if a participant made a response after 880 ms in a trial, 9 dots were shown in that trial (onset of the 9th dot was at 800 ms). If we are interested in the time point 120 ms after dot onset (dot position change), this gives us 8 time points within that trial that were 120 ms after a dot onset. Further excluding all time points 200 ms before the response and later, would leave us with 6 data points for this example trial, see Figure 1 for an illustration. For each time after dot onset and for each participant we concatenated all of these data points across trials to one long vector and inferred regression coefficients on these expanded data sets. Note that this approach can equally be interpreted as statistical inference over how strongly the sequence of momentary evidence caused by the dot updates is represented in the signal at 100 ms wide steps with a delay given by the chosen time from dot onset.

These analyses included two regressors of interest: momentary evidence (x-coordinate) and y-coordinate of the associated dots. We additionally included the following nuisance regressors: an intercept capturing average effects, the absolute values of x- and y-coordinates, perceptual update variables for x- and y-coordinates (Wyart et al., 2012) defined as the magnitude of the change from one dot position to another and accumulated values of x- and y-coordinates. Other than the standard regression analysis, the expanded regression analysis did not include the response as nuisance regressor, because the expanded regression analysis specifically models within-trial changes while the response is fixed throughout each trial. Because we found that the accumulated values can be strongly correlated with the individual x- and y-coordinates (cf. Figure S1), we only used accumulated values up to the previous dot in the regressor. For example, if a data point was associated with the x-coordinate of the 4th dot, the accumulated regressor would contain the sum of only the first three x-coordinates. This accumulated regressor is equal to the regressor resulting from Gram-Schmidt ortho-normalization of the full sum of x-coordinates with respect to the last shown x-coordinate. The accumulated evidence regressor was derived from the ideal observer model as the log posterior odds of the two alternatives, but this was almost 100% correlated with the simple sum of x-coordinates. The small differences between model-based accumulated evidence and sum of x-coordinates after normalization resulted from a small participant-specific offset representing the overall bias of the participant toward one decision alternative. Note that we do not show any results for this (previous) accumulated evidence regressor.

In Figures 6, 8 we report results from separate expanded regression analyses in which we replaced the x-coordinate regressor with the sum of x-coordinates and dropped the previous accumulated evidence regressor. We did this, because the previous accumulated evidence regressor did not allow us to estimate effects of accumulated evidence for the first 100 ms after dot onset which is possible with the separate regression. We also did not see any benefits from using the previous accumulated evidence regressor in comparison to the simple sum of x-coordinates up to the current dot. Although the previous accumulated evidence regressor is in principle Gram-Schmidt orthogonalized with respect to the current, i.e., last presented x-coordinate and therefore provides independent information from the current x-coordinate, this is not the orthogonalization that we are most interested in. Ideally we would want to orthogonalize with respect to any information about x-coordinates, i.e., momentary evidence including information contributed by the whole series of x-coordinates. So, while the previous accumulated evidence regressor is orthogonal to the current x-coordinate, it still correlates with the x-coordinates of previously presented dots. As accumulated evidence is just the sum of x-coordinates, this cannot be prevented so that momentary and accumulated evidence regressors will always partially capture overlapping effects. We still found it informative to present a separate analysis for accumulated evidence under the premise that the effects of the accumulated evidence regressor more strongly relate to accumulated evidence than momentary evidence and vice-versa for the momentary evidence regressor.

##### 2.5.1.3. Response-aligned regression analyses

Additional to the first-dot onset and dot onset aligned analyses, we conducted response-aligned analyses in which time was referenced to trial-specific response times of participants. The regressors in this analysis were the trial-specific choice of the participant, trial-time and an intercept. Choice was encoded as –1 for left and +1 for right so that the direction of correlations was compatible with that for the evidence regressors. The trial-time regressor simply counted the trial number within the experiment per participant. Timed out trials were excluded from analysis. As in the other regression analyses we z-scored regressors and data across trials before estimating the regression coefficients, except for trial-time which was only scaled to standard deviation equal to 1.

We performed two different analyses in sensor and source space. In sensor space (magnetometers) we ran independent univariate regressions for each combination of sensor and time so that we ran 102 ^*^ 70 regressions with maximally 480 data points (one per trial, minus excluded trials). We report results of this analysis in Figure 9.

After having identified time windows of interest based on the sensor level results, we estimated the mean regression coefficients across the selected time window for each brain area and participant. To do this, we aggregated data from the identified times into a common regression on source data. We concatenated the data from all times in the time window and performed the regression on this expanded data set, then including maximally number of trials ^*^ number of time points data points. We report results of this analysis in Figure 10.

##### 2.5.1.4. Normalization

The regression analyses described above used normalization of data and regressors to produce coefficients that can be interpreted as approximate measures of correlation. Specifically, we normalized data (sensor values or estimated source currents) to have mean 0 and standard deviation 1 (z-score) across trials, but within time points and participants. In expanded regression analyses this normalization was conducted before combining data from single time points into one regression analysis. We also z-scored the individual regressors of the regression analyses (except for the constant intercept). We conducted this normalization directly on the constructed, participant-specific design matrices that were passed to the optimization procedure solving the regression model, i.e., in expanded regression analyses this was after the data of single time points from one participant was combined into one regression model. We say that this normalization leads to approximate correlation coefficients, because it can be shown that the coefficients resulting from a least-squares fit of a linear regression with z-scored data and z-scored regressors are equal to Pearson correlation coefficients between data and regressors, if the z-scored regressors are perfectly uncorrelated. For further information about normalization see Supplementary Data Sheet 2.

#### 2.5.2. Identification of Significant Source-Level Effects

To identify significant correlations between regressors of interest and source signals we followed the summary statistics approach (Friston et al., 2006) and performed two-sided *t*-tests on the second level (group-level, *t*-tests across participants). We corrected for multiple comparisons across time points and brain areas by controlling the false discovery rate using the Benjamini-Hochberg procedure (Benjamini and Hochberg, 1995). Specifically, for identifying significant effects reported in Figure 7 we corrected across 25,340 tests covering 70 time points (0 to 690 ms from dot onset in 10 ms steps) and 362 brain areas (180 brain areas of interest per hemisphere plus one collection of sources per hemisphere that fell between the area definitions provided by the atlas). We report all significant effects of this analysis in Supplementary Data Sheet 1.

#### 2.5.3. Identification of Significant Differences in Correlation Patterns

We formally investigated the differences in correlation patterns of the response-aligned analysis between the two time windows of interest (Figures 9, 10). As we were interested in the differences between spatial patterns, we accounted for the overall increase in correlation magnitudes from the build-up to the response window by normalizing the correlation magnitudes. This normalization consisted of first shifting the minimum magnitude to 0 and then scaling the resulting magnitudes so that their mean equals 1 across sensors or brain areas. The shift of the magnitudes prevents excessive shrinking of magnitude variances for magnitude patterns with overall large magnitudes and ensures that the magnitudes have similar distributions across the involved sensors or brain areas in both considered time periods. We subsequently computed the differences between the selected time periods on the first level and report second-level (across participant) statistics.

The difference topography in Figure 9 directly shows the computed mean difference. Additionally we applied a *t*-test across participants within each sensor, corrected the resulting p-values for false discovery rate at α = 0.01 across sensors and indicate the resulting significant effects with white dots.

The analysis on the source level was in principle equal to the one on the sensor level, but in addition accounted for the fact that most brain areas were not involved in encoding the choice. We achieved this by computing the normalization parameters for a time window only across brain areas with a significant effect in this time window. We then computed magnitude differences for all brain areas with a significant effect in at least one of the time windows and proceeded with second-level statistics for these areas as before.

### 2.6. Code Accessibility

Code implementing the statistical analysis which produced all presented results is available at https://github.com/sbitzer/BeeMEG.

## 3. Results

**Figure 2:**
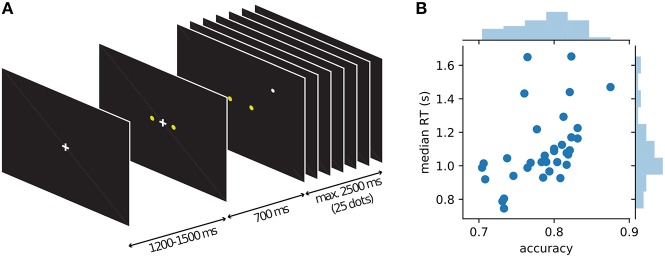
Course of events within a trial in the single dot task and behavior of individual participants. **(A)** Each trial started with the presentation of a fixation cross, followed by the appearance of the two yellow targets after about 1 s. 700 ms after the appearance of the targets the fixation cross disappeared and a single white dot was presented at a random position on the screen (drawn from a 2D-Gaussian distribution centered on one of the targets). Every 100 ms the position of the white dot was changed to a new random draw from the same distribution. Participants were instructed to indicate the target which they thought was the center of the observed dot positions. After 25 dot positions (2.5 s) without a response, a new trial was started automatically, otherwise a new trial started with the response of the participant. **(B)** Average behavior (accuracy and median response time) for each of the 34 participants shown as blue dots and histograms.

While MEG was recorded, 34 human participants observed a single white dot on the screen changing its position every 100 ms and had to decide whether a left or a right target (two yellow dots) was the center of the white dot movement (Figure 2). Under moderate time pressure (see Methods), participants indicated their choice with a button press using the index finger of the corresponding hand. The distance of the target dots on the screen was chosen in behavioral pilots so that participants had an intermediate accuracy around 75% while being told to be as accurate and fast as possible. The average median response time across participants was 1.1 s with an average accuracy of 78% (cf. Figure 2B).

Our paradigm dissociates two different kinds of information available to the participants from the stimulus. The x-coordinates of the jumping white dot convey decision-relevant perceptual information while the y-coordinates convey perceptual information that is irrelevant for the decision. We assume that both signals are processed by the brain, but only the decision-relevant x-coordinates are taken into account when making a decision.

To define decision evidence, we used a computational model. An ideal observer model for inference about the target given a sequence of single dots has been described before (Bitzer et al., 2014; Park et al., 2016). This model identifies, as expected, the x-coordinates of the white dot positions as momentary decision evidence. Specifically, there is a direct linear relationship between x-coordinates and momentary evidence so that in the following regression analyses we could directly use the x-coordinates as independent variables instead of having to compute decision evidence from the x-coordinates through the model (see Methods). We further identified the cumulative sum of x-coordinates across single dot positions as accumulated evidence which corresponds to the average state of a discrete-time drift-diffusion model (Bitzer et al., 2014).

### 3.1. Participants Integrate Evidence Provided by Single Dot Positions to Make Decisions

**Figure 3:**
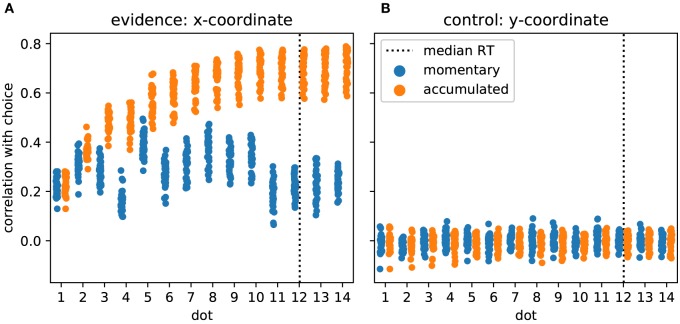
Participants accumulate momentary evidence provided by dot positions for making their decisions. **(A)** Each shown point corresponds to the Pearson correlation coefficient for the correlation between choices of a single participant and the sequence of presented dot positions across the 480 trials of the experiment. We plot, over stimulus duration, the momentary (blue) and the accumulated evidence (orange). The dotted vertical line shows the median RT across participants. Until about the 10th dot presentation the correlation between accumulated evidence and participant choices rises, reaching values around 0.7 while the momentary evidence is only modestly related to participant choices across all dots. **(B)** The same format as in A but all measures are computed from the y-coordinates of dot positions which were irrelevant for the decision. As expected, y-coordinates do not correlate with participant choices.

As the task required and the model predicted, participants made their decision based on the provided evidence. In Figure 3 we show this as the correlation of participants' choices with momentary and accumulated evidence. Momentary evidence was mildly correlated with choices throughout the trial (correlation coefficients around 0.3) while the correlation between accumulated evidence and choices increased to a high level (around 0.7) as more and more dot positions were presented. This result indicates that participants accumulated the momentary evidence, here the x-coordinate of the dot, to make their choices. In contrast, as expected, the y-coordinates had no influence on the participants' choices as indicated by correlation coefficients around 0 (Figure 3B).

Previous work has investigated the influence of individual stimulus elements on the eventual decision and whether this influence differed across elements (Wyart et al., 2012; Hubert-Wallander and Boynton, 2015). In our analysis this corresponds to checking whether the correlations with momentary evidence shown in Figure 3A differ across dots. This is clearly the case [*F*_(13, 462)_ = 65.49, *p* ≪ 0.001]. Contrary to previous work (Hubert-Wallander and Boynton, 2015) we do not observe a primacy effect. Instead, we observe a particularly large difference in the influence of the 4th and 5th dots on the decision (*post-hoc* paired *t*-test: *t*(33) = –34.90, *p* ≪ 0.001 with the 5th dot having a strong influence while the 4th dot having a relatively small influence. This reflects our pre-selection and manipulation of stimuli which were partially chosen from a previous experiment to induce large response times (leading to the small influence of the 4th dot) and a manipulation of the 5th dot to create large variation in x-coordinates (see Methods for further details). Taken together these results confirm that the used stimuli were effective in driving the decisions of the participants and that the theoretically defined momentary and accumulated evidence integrate well with observed behavior.

### 3.2. MEG Signals Covary With Momentary Evidence at Specific Time Points After Stimulus Update

For the analysis of the MEG data we used regression analyses computing event-related regression coefficients of a general linear model (Hauk et al., 2006; Clarke et al., 2013). For our main analysis the regressors of interest were the momentary evidence and, as a control, the y-coordinates of the presented dots. We normalized both the regressors and the data so that the resulting regression coefficients can be interpreted as approximate correlation values while accounting for potential covariates of no interest (see Methods). Note that this correlation analysis is different from standard event-related field analyses, where one would only test for the presence of a constant time-course across trials. With the correlation analysis, the estimated regression coefficients describe how strongly the MEG signal, in each time point and each sensor (or source), followed the ups and downs of variables such as the momentary evidence, across trials.

**Figure 4:**
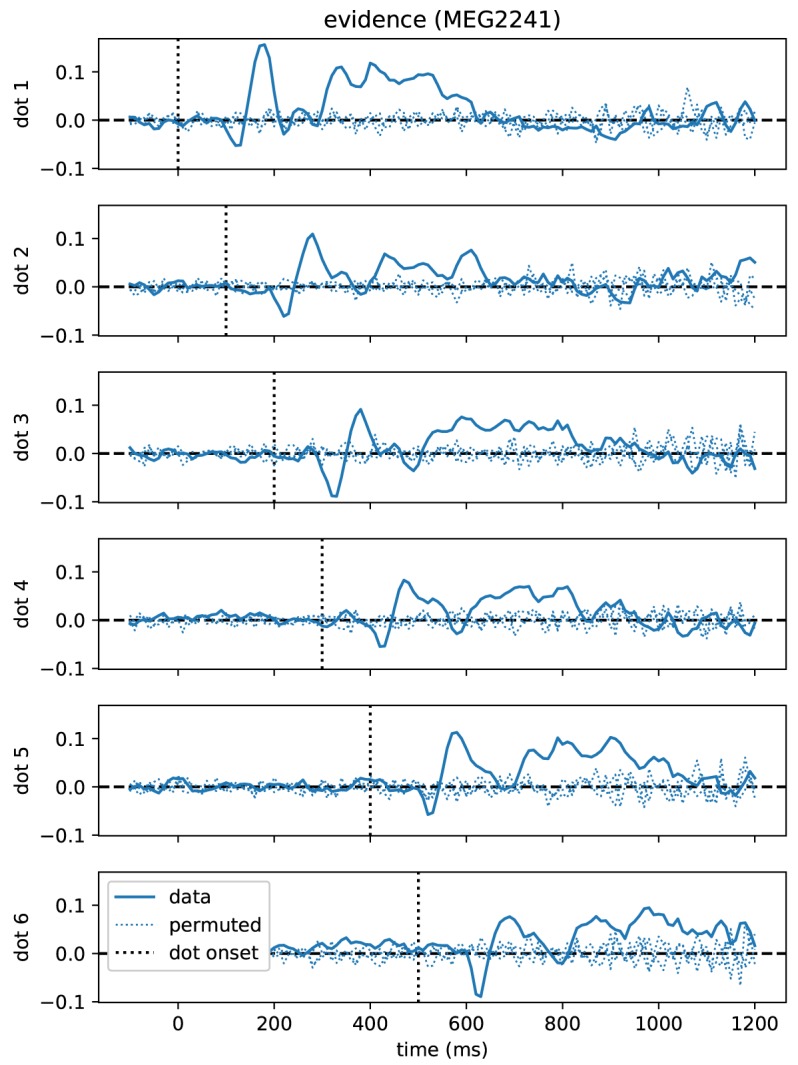
Time course of correlations with momentary evidence repeats for each dot shifted by dot onset times. In the standard regression analysis there was one regressor for each element in the sequence of dot positions (dots). This allowed us to test, when after first dot onset, correlations with the considered dot could be observed. The figure demonstrates, for the magnetometer channel with the strongest average correlations, that the correlation time course exhibits roughly a stereotyped profile relative to the onset time of the dot on the second level (average across subjects, grand average). Dotted lines show the same quantity, but for data that we permuted over trials before the regression analysis.

As a first result, we found that correlations between momentary evidence and MEG signals followed a stereotypical temporal profile after each dot position update (Figure 4). Therefore, we performed an expanded regression analysis where we explicitly modeled the time from each dot position update, which we call “dot onset” in the following. To exclude the possibility that effects from the button press motor response influenced the results of the dot onset aligned analysis, we only included data, for each trial, up until at most 200 ms prior to the participant's response.

**Figure 5:**
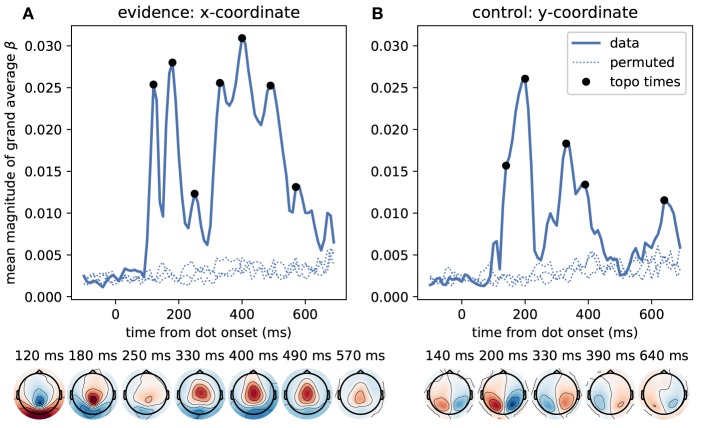
Time course of correlation strengths between magnetometer measurements and momentary evidence and perceptual control variable. **(A)** The top panel shows the time courses of the mean (across sensors) magnitude of grand average regression coefficients (β). For comparison, dotted lines show the corresponding values for data which were randomly permuted across trials before statistical analysis. Black dots indicate time points for which the sensor topography is shown below the plot. These topographies directly display the grand average regression coefficients at the indicated time without rectification, i.e., with negative (blue) and positive (red) correlation values. The momentary evidence has strong correlations with the magnetometer signal at 120, 180 ms and from about 300 to 500 ms after dot onsets. **(B)** The correlations with the decision irrelevant y-coordinate are visibly and significantly weaker than for the evidence, but there are two prominent peaks from about 120 to 210 ms and at 320 ms after dot onset. There is no sustained correlation with the y-coordinate beyond 400 ms and the topographies of magnetometers differ strongly between evidence and y-coordinates. Specifically, the evidence exhibits occipital, centro-parietal and central topographies whereas the y-coordinate exhibits strong correlations only in lateral occipito-parietal sensors.

We first identified time points at which the MEG signal correlated most strongly with the momentary evidence. For these sensor-level analyses we used magnetometer sensors only. We performed separate regression analyses for each time point from dot onset, magnetometer sensor and participant, computed the mean regression coefficients across participants, took their absolute value to yield a magnitude and averaged them across sensors. Figure 5 shows that the strongest correlations between momentary evidence and magnetometer signals occurred at 120 ms, 180 ms and in a prolonged period from roughly 300 to 500 ms after dot onset. In contrast, correlations with the decision irrelevant control variable, that is, the dot y-coordinates, were significantly lower in this period from 300 to 500 ms (two-tailed Wilcoxon test for absolute average coefficients across all sensors and times within 300–500 ms, *W* = 382781, *p* ≪ 0.001).

The sensor topographies shown in Figure 5 indicate for the momentary evidence a progression of the strongest correlations from an occipital positivity over a centro-parietal positivity to a central positivity. y-coordinate correlations, on the other hand, remained spatially at occipito-parietal sensors.

### 3.3. Correlations With Accumulated Evidence

Guided by the model we used dot x-coordinates as representation of momentary evidence, but dot x-coordinates also do have a purely perceptual interpretation similar to the y-coordinates as they simply measure the horizontal location of a visual stimulus. Correlations with x-coordinates, therefore, may reflect at some time points early visual processes independent of the decision, at some time points momentary evidence and other time points both of them. Contrasting the strength of significant effects for x- and y-coordinates (Figure 5) already suggested that at least from 400 ms after dot onset x-coordinates indeed represented a form of decision evidence. Here we further corroborate this finding by turning to a form of decision evidence that has no direct purely perceptual interpretation and is more closely related to the decision itself: the accumulated evidence.

The accumulated evidence is, through the final choice, more strongly related to the motor response than the momentary evidence (cf. Figure 3A, Figure S1). To account for this potential confound we excluded, as before, all data later than 200 ms before the response so that the results only contain effects unrelated to the actual motor response.

**Figure 6:**
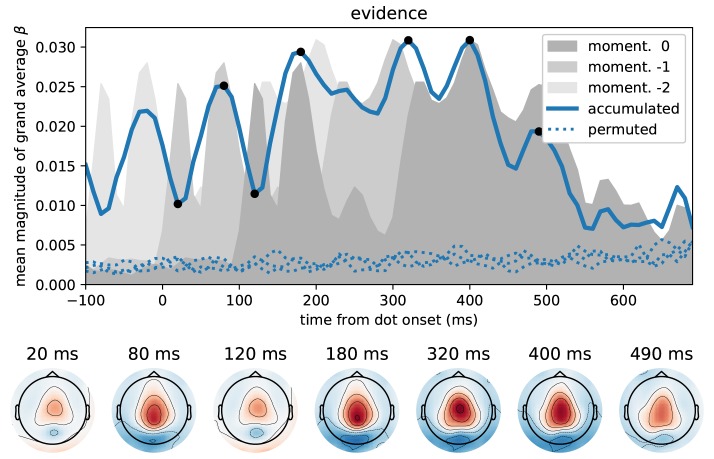
Correlation of accumulated evidence and magnetometer signals relative to dot onset display central sensor topographies. Format as in Figure 5, i.e., the blue line in the top panel shows the time course of the mean (across sensors) magnitude of grand average regression coefficients (β) together with corresponding time courses after 3 different permutations across trials (dotted). In addition, we show the momentary evidence time course (dark gray shade, cf. Figure 5A) and two replica of it which are shifted by 100 ms to the past (lighter grays). These time courses, therefore, are associated with the representation of the momentary evidence/x-coordinates of the current dot, the previous dot and the dot before that in the brain.

Figure 6 depicts the time course of overall correlation magnitudes for accumulated evidence together with effect topographies at chosen time points. We found significant correlations between the MEG signal and accumulated evidence at all peri-stimulus times until about 550 ms after dot onset. Crucially, during this time period we observed centro-parietal and, especially, central sensor topographies suggesting that these represent specifically decision-relevant information such as momentary or accumulated evidence, as hypothesized based on the correlations with x-coordinates shown in Figure 5.

While the brain may represent momentary evidence only transiently until it is included in the accumulated evidence, accumulated evidence should, by definition, be represented more persistently. Except for the two dips at 20 and 120 ms, the correlation time course shown in Figure 6 indeed suggests that accumulated evidence is represented in the MEG signals more persistently than the momentary evidence. In fact, The early dips in the accumulated evidence correlations are likely artifacts resulting from an interaction with ongoing perceptual processing of the individual input stimulus (x-coordinate of white dot) that we observe as the 120 ms peak in momentary evidence correlations (Figure 5). To make this clear, we overlayed the correlation time course of the accumulated evidence with that of the momentary evidence from Figure 5, along with a time-shifted replica of the momentary evidence. The two momentary evidence time courses visualize the times at which the MEG signals correlated strongly with the x-coordinates of the white dot shown from 0 to 100 ms, as well as the previous dot shown from –100 to 0 ms (light gray replica in Figure 6). Figure 6 therefore shows that the early dips in accumulated evidence correlations coincide with the early 120 ms peaks of each of the momentary evidence correlations. Furthermore, comparing the associated topographies in Figures 5, 6, we see that the momentary evidence has a central negativity at 120 ms, while accumulated evidence has a central positivity throughout the time window of interest. The two representations of momentary and accumulated evidence in the MEG therefore correspond to opposite effects which cancel, when they co-occur. This apparently happens during the early perceptual processing stage of a dot around 120 ms after its onset resulting in a lowered correlation with the accumulated evidence at these time points.

In conclusion, these results suggest that the correlations with accumulated evidence indeed are a representation of accumulated evidence and not momentary evidence. Further this representation appears to manifest itself with a central positivity in the MEG magnetometers.

### 3.4. Sources of Stimulus-Aligned Momentary Evidence Effects

By investigating the sources of the evidence correlations at sensor level, we aimed to better understand the nature of these effects and to confirm their locations in the brain suggested by the shown sensor topographies. In particular, we were interested in linking the time points for which we found strong momentary evidence correlations to potential functional stages in the processing of decision evidence, such as sensory processing, relating sensory information to the decision and integrating momentary evidence with previous evidence.

We reconstructed source currents along the cerebral cortex for each participant and subsequently repeated our regression analysis on the estimated sources (see Methods and Materials for details).

**Figure 7:**
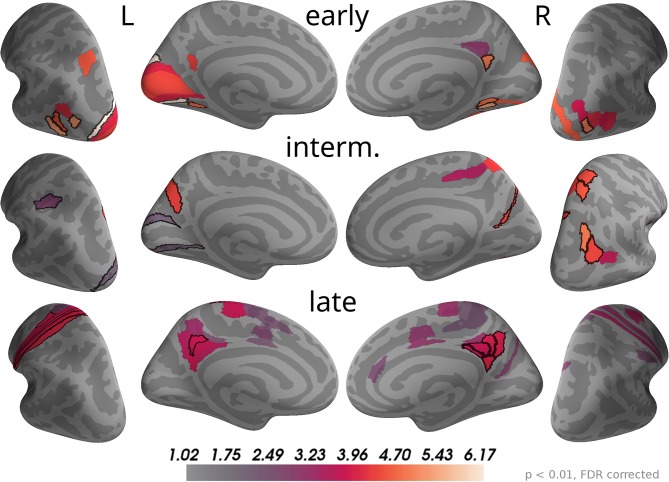
Correlations with momentary evidence shift from visual over parietal to motor and posterior cingulate areas. We investigated the three time windows with strong correlations in the sensor-level results: early (110–130 ms), intermediate (160–200 ms), and late (290–510 ms). For each of these phases only brain areas with at least one significant effect (*p* < 0.01, FDR corrected) within the time window are colored. For display purposes, colors show average second-level *t*-value magnitudes where the average is taken over time points within the time window. The 5 areas with the most consistent, strong correlations per hemisphere and time window are marked by black outlines. These were (in that order; specified as Brodmann areas with subdivisions as defined in Glasser et al., 2016): early, left—V3, FST, LO3, VMV2, MST; right—VMV2, LO1, v23ab, VMV1, VVC; intermediate, left—POS2, AIP, V2; right—IP0, VIP, 7AL, PGp, DVT; late, left—1, 3a, 6d, 3b, 31pd; right—v23ab, 7m, 31pd, 31pv, d23ab.

The time course of correlation magnitudes shown in Figure 5 suggested three time windows at which particularly strong correlations with momentary evidence were present in the brain. The source analysis gives equivalent results: Multiple comparison corrected effects occurred only within 110–130 ms, 160–200 ms, and 29–510 ms (cf. Supplementary Data Sheet 1). In subsequent analyses we, therefore, focused on these time windows and call them according to their temporal order "early," "intermediate," and "late" phases. Figure 7 depicts the brain areas with at least one significant multiple comparison corrected effect within the corresponding phase. The color scale indicates the average *t*-value magnitudes within the time window for these significant areas (we chose to display *t*-value magnitudes instead of correlation magnitudes here, because the estimated correlation values had larger second-level variability differences across brain areas than sensors).

As the sensor topographies suggested, we observed that in the early phase the strongest correlations were located in visual areas such as V3, V1 and areas in the lateral occipital cortex (e.g., FST, MST, LO3 according to Glasser et al., 2016), but also in a small area of posterior cingulate cortex (v23ab) and there was an effect in a parietal area of the left hemisphere (MIP). In the intermediate phase most of the correlations in visual areas, especially those in lateral occipital areas, vanished. Instead, more parietal areas exhibited significant correlations with momentary evidence, especially in the right inferior (IP0, PGp) and superior parietal cortex (VIP, 7AL, 7Am). Additionally, we found strong correlations in posterior cingulate cortex (POS2 and DVT). In the late phase some correlations in parietal areas persisted, but only focal at some time points so that on average across the time window correlations were weak compared to other brain areas. Specifically, the strongest correlations were spread across the posterior cingulate cortex in both hemispheres (especially areas v23ab, 31pd, 7m, 31pv, d23ab). Further strong correlations occurred in motor areas, especially in the left hemisphere, including somatosensory areas (3a, 3b, 1), primary motor cortex (area 4) and premotor areas (6a, 6d). Note that we excluded from the analysis all time points later than 200 ms before the trial-specific motor response. Additionally, we observed weaker correlations in mid and anterior cingulate motor areas (e.g., 24dv, p24pr).

These results confirm that the information carried by the decision-relevant x-coordinates shifts from visual over parietal areas toward motor areas. Given that the total number of time points with significant momentary evidence correlations was larger for motor regions than for parietal regions, it appears that parietal areas represented momentary evidence for a shorter duration than motor areas. As this was no central question in the present work, we did not further substantiate this finding with a formal statistical analysis so that we cannot draw definite conclusions about its validity.

The results also reveal that source currents of brain areas in posterior cingulate cortex had strong correlations with x-coordinates throughout all three phases. Accordingly, the areas with the largest correlation magnitudes on average across all time points within 0 to 500 ms were predominantly located in posterior cingulate cortex (5 areas with strongest average effects in that order: left—v23ab, 3a, 31pd, 3b, 1; right—v23ab, DVT, d23ab, 31pv, 7m). This suggests a potentially central role of posterior cingulate cortex in the processing of momentary evidence in the task.

### 3.5. Sources of Stimulus-Aligned Accumulated Evidence Effects

The sensor topographies for the accumulated evidence effects suggested that accumulated evidence was represented in common brain sources across the whole time window of 0 to 550 ms from dot onset. Therefore, we used this full time window to investigate the underlying sources. As for the momentary evidence, cf. Figure 7, we identified brain areas with significant correlations after FDR correction across locations and times (*p* < 0.05, no significant effects for *p* < 0.01) in at least one time point and then averaged the *t*-value magnitudes across time points within the time window in these areas. Given the similarity of sensor topographies of momentary evidence in the late phase and the sensor topographies of accumulated evidence we expected their sources to overlap.

**Figure 8:**
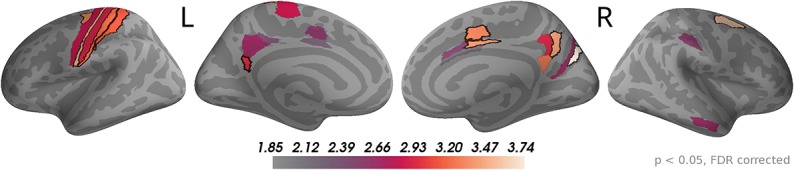
Sustained correlations with accumulated evidence in motor and cingulate areas. Following the procedure in Figure 7, we colored only areas with a significant correlation with accumulated evidence (*p* < 0.05 FDR corrected) with color indicating the average *t-*value magnitude in the extended time window from 0 ms to 550 ms after dot onset. The 5 largest effects were (marked by black boundaries): left—3a, 6d, 1, 2, v23ab; right—V6, 6a, 7m, p24pr, 24dv.te.

In Figure 8, one can see that, although the estimated correlation magnitudes were slightly higher for the accumulated evidence than for the momentary evidence, fewer effects were statistically significant for accumulated evidence. This is most likely because the variability of correlation magnitudes across participants increased relative to momentary evidence effects (results not shown). Otherwise, the identified brain areas were consistent with those of the momentary evidence in the late phase. In particular, we observed consistently strong correlations with accumulated evidence in motor, premotor, cingulate motor, and posterior cingulate areas.

### 3.6. Correlations With Choice Reveal Response-Aligned Build-Up and Separate Motor Response

Our finding that momentary or accumulated evidence is represented in motor areas is consistent with a wide range of previous work (Donner et al., 2009; Michelet et al., 2010; Selen et al., 2012; de Lange et al., 2013; Kelly and O'Connell, 2013; Thura and Cisek, 2014). If motor areas are involved in processing momentary or accumulated evidence prior to a response, as these results indicate, the question arises how these processes relate to motor processes linked to the response itself. More specifically, we were interested in how the patterns of correlations with momentary and accumulated evidence related to correlation patterns representing the motor response and whether these could be linked to the absence or presence of the involvement of certain brain areas. To investigate correlation patterns representing the motor response we computed choice-dependent effects centered on the response time of the participants. We did this with a regression analysis using the participant choice as a regressor of interest (see Methods). The choice regressor provides a measure for how well the choice of the participants can be decoded from univariate brain signals.

**Figure 9:**
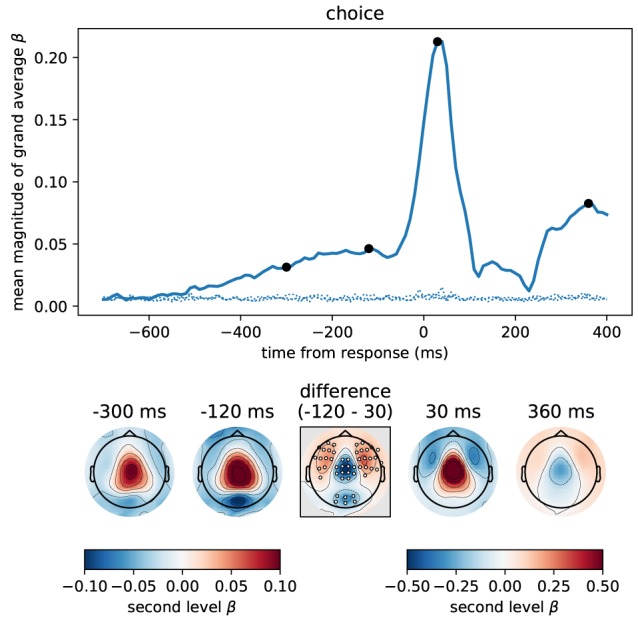
The button press motor response peaking at 30 ms is represented most strongly in central magnetometers, but the corresponding topography differs slightly from that associated with momentary and accumulated evidence. We computed the correlation between participant choices and MEG magnetometers using linear regression for data aligned at response time. Following the format of Figure 5 we here show the time course of the mean (across sensors) magnitude of grand average regression coefficients (β). Sensor topographies for time points indicated by the black dots are shown below the main panel. Note that for the time points before the response we use a different scaling of colors than for time points around the response and later. This is to more clearly visualize the topography around the response which contains larger values. The color scaling for the time points before the response is equal to that of Figures 5, 6. The topography at –300 ms strongly resembled that for accumulated evidence, but the topography around the response (30 ms) additionally exhibited stronger fronto-lateral and weaker occipital anti-correlations (*p* < 0.01 corrected, cf. middle difference topography, see Methods for details). Positive values / correlations mean that measured sensor values tended to be high for a right choice (button press) and low for a left choice and vice-versa for negative values.

Figure 9 depicts the estimated time course of correlation magnitudes averaged across participants and sensors. From about 500 ms before the response, correlations between choice and MEG data became gradually stronger culminating in an expected peak centered slightly after the response. The sensor topographies of the build-up period before the response strongly resembled those we found for accumulated evidence in our previous analyses. In fact, these results most likely correspond to the same effect, because the participant choice itself was increasingly correlated with accumulated evidence as the trial progressed (cf. Figure 3). That is, the build-up seen in the figure only indirectly visualizes an increasing evidence signal by depicting an increasing alignment of the final choice with the internal representation before the response (presumably accumulated evidence).

The motor response itself (peak around 30 ms) was, as expected, much more strongly represented in the MEG signals than the accumulated evidence, see Figure 9. Although the motor response also had a predominantly central topography, its topography visibly differed from that prior to the response (at –300 and –120 ms). Specifically, the topography before the response exhibited stronger anti-correlation in occipital sensors than around the response while the topography around the response exhibited stronger anti-correlations in fronto-lateral sensors (*p* < 0.01 corrected, cf. Figure 9, difference topography). Furthermore, the correlation with choice was relatively higher over central sensors at 30 ms than at –120 ms (Figure 9, difference topography).

**Figure 10:**
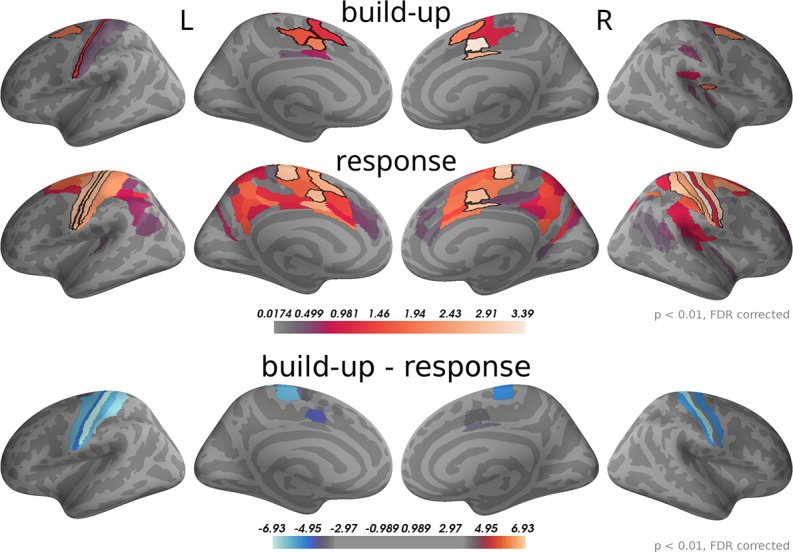
Around the response time strongest correlations with choice occurred in primary motor, somatosensory and cingulate motor cortex (BA 24) while during the build-up period (–500 to –120 ms) we found the strongest effects in premotor and cingulate motor cortex. The 5 largest effects per hemisphere were: build-up, left—24dv, 6a, 24dd, 3a, SCEF; right—24dv, p24pr, 6a, SCEF, OP2-3; response, left—4, 3b, 24dv, 3a, SCEF; right—3b, 4, p24pr, 24dv, 2. When testing for differences in the spatial pattern of correlation magnitudes (see Methods) between the two time windows, we only found significant differences in the motor and cingulate areas: 1, 24dv, 2, 31a, 3a, 3b, 4, 6d, 6mp, SCEF, p24pr. All of these effects indicated that correlations with choice were stronger in the response window (blue). The build-up and response panels show spatially normalized *t*-value magnitudes while the difference panel shows *t*-values of spatially normalized correlation magnitude differences.

To analyse this difference at the source level we applied the regression analysis to the reconstructed source currents. Figure 10 depicts the results of an analysis of two time windows: the "build-up" window from –500 to –120 ms (when a dip before the response indicates an end of the build-up) and the "response" window capturing the response peak from –30 ms to 100 ms. We only show brain areas with at least one significant effect within the time window after correcting for multiple comparisons (FDR with α = 0.01 across brain areas and the two time windows). The shown colors indicate normalized second-level *t*-value magnitudes (see Methods).

As expected, in the response window, the effects were dominated by choice correlations in bilateral primary motor and somatosensory cortices, but also choice correlations in cingulate motor areas (around Brodmann area 24) were among the effects with the strongest magnitudes. Other significant correlations with choice within the response window occurred in premotor and posterior cingulate cortices. In the build-up window, the strongest correlations occurred predominantly in cingulate motor cortex and premotor areas (especially 6a).

We further aimed at identifying brain areas with significantly different correlation magnitudes in the two time windows. Specifically, we were interested in the difference of the spatial patterns of correlation magnitudes, across brain areas, between the two time windows. To do this, we normalized correlation magnitudes across brain areas within the time windows and computed the differences between time windows within each brain area and participant (see Methods for details). Figure 10, bottom panel, shows that across participants the only statistically significant differences occurred in the primary motor and somatosensory cortices and, with smaller effect size, in cingulate motor areas. In all these areas correlation magnitudes were larger in the response as compared to the build-up window.

In summary, the response-centered analysis of choice correlations suggests that the build-up of choice-correlations leading toward a response is related to the accumulation of momentary evidence, because sensor topographies and brain areas were highly consistent across choice- and evidence-based analyses. The correlation topographies for the build-up and the response windows shown in Figure 9 had significant differences in central, occipital and fronto-lateral sensors. When analysing these differences at the source level (Figure 10), the only sources with significant differences were located in motor areas. These results together suggest that the brain areas representing decision evidence are largely overlapping with those representing the upcoming choice and the motor response. The difference in correlation patterns at the source level between the upcoming choice and motor response may be explained by an increase in choice correlations in motor areas.

## 4. Discussion

Using MEG, we analyzed the dynamics of evidence representations in the human brain during perceptual decision making. We induced fast, within-trial evidence fluctuations in which new evidence appeared every 100 ms and correlated the resulting momentary evidence dynamics with MEG signals. We identified three main phases of the representation of momentary evidence: an early phase around 120 ms post evidence update, an intermediate phase around 180 ms and a late phase from about 300 to 500 ms. These phases exhibited different sensor topographies with positive correlations shifting from occipital to centro-parietal to central sensors during the three phases. We localized the sources of these representations in early visual, parietal and motor areas respectively, with significant correlations in posterior cingulate cortex occurring in all three phases. Significant correlations with accumulated evidence occurred continuously until about 550 ms after update onset and exhibited a central topography similar to that in the late phase of momentary evidence representations with corresponding sources. Additionally, correlations with response-aligned MEG signal shared a similar topography with a build-up phase hundreds of milliseconds before the response. Further analysis showed that the only significant differences between build-up phase and motor response were higher choice correlations in motor areas during response.

It has previously been shown that the MEG signals correlate with individual pieces of momentary evidence (de Lange et al., 2010; Gould et al., 2012; Wyart et al., 2012; Gluth et al., 2013). In contrast to these studies we used a rapid evidence fluctuation paradigm (10 Hz) presumably closer to natural settings. More importantly, we track momentary evidence representations and the corresponding areas in the human brain through the three phases that we newly identified, although at least the early and late phases were previously hinted at (Wyart et al., 2012).

There is overwhelming evidence that motor areas including the premotor and primary motor cortex are involved in perceptual decision making (Heekeren et al., 2008; Hanks and Summerfield, 2017). Specifically, it has been shown that some single neurons in primary motor cortex represent momentary evidence (Thura and Cisek, 2014), that motor-evoked potentials can be related to accumulated evidence (Michelet et al., 2010; Hadar et al., 2016) and that classical lateralized readiness potentials (Smulders and Miller, 2012) also exhibit evidence-dependent build-up in a detection task (Kelly and O'Connell, 2013), and single-trial pre-movement MEG activities are correlated with reaction times in the motor area (Smyrnis et al., 2012). Our results further substantiate these findings by showing that human motor areas represent each update of momentary evidence roughly within 300 to 500 ms after the update onset and that accumulated evidence is represented in motor areas throughout the decision making process. By analysing response-aligned data to detect choice-dependent effects, we further showed that the stimulus-aligned evidence representations resemble closely the representation of the final choice during a build-up phase before the response. This supports the hypothesis that observations of pre-response representations of an upcoming choice, such as the lateralized readiness potential, should be interpreted as expressions of an ongoing decision making process about the next sensible motor response. In sum, the present and previous findings affirm a tight coupling between decision making and motor processes, as formulated in the affordance competition hypothesis (Cisek, 2007; Cisek and Kalaska, 2010), and also in other cognitive computational neuroscience theories (O'Regan and Noë, 2001; Friston et al., 2009; Clark, 2013).

One potential caveat of our correlation results in motor areas is that participants may have executed micro-movements to track the changes of the stimulus either with their eyes, or with minimal finger movements before response. In this scenario the observed correlations in motor areas could be explained by motor signals to the muscles. Although we cannot completely exclude this possibility we deem it unlikely; (i) Most stimuli were within 5° diameter from fixation meaning that most of them were well within the foveal visual field. (ii) The sensor topographies representing evidence were very similar to that associated with the motor response, that is, the evidence representations do not appear to be specifically related to eye movements. (iii) As mentioned above, a large body of work already supports the interpretation that motor areas represent decision evidence before motor execution. In conclusion, we do not believe that the correlations observed in motor areas of the brain are merely an expression of motor control signals that caused stimulus-correlated micro-movements. Even if such micro-movements existed, these would most likely follow the time-course of decision evidence rather than decision-irrelevant stimulus properties (Michelet et al., 2010; Selen et al., 2012; Hadar et al., 2016).

Although we found some representations of momentary evidence in the parietal areas during the intermediate and late phase, the strongest representations were found predominantly in the motor or somatosensory, and cingulate areas. This was also the case for accumulated evidence. These results suggest that in our task the parietal cortex was not the main area involved in the processing of momentary evidence and did not appear to accumulate evidence for the decision, or at least did not represent accumulated evidence over an extended period of time.

These findings appear to be at odds with previous observations in non-human primates which had identified neurons in inferior parietal cortex that seemed to represent accumulated evidence (Gold and Shadlen, 2007). More recent work, however, suggests that the firing of these neurons is more diverse than originally thought (Meister et al., 2013; Park et al., 2014; Latimer et al., 2015). It is therefore possible that the signal from only few evidence accumulating neurons in inferior parietal cortex is too weak to be recorded with MEG. Another possibility is that the representation of decision evidence in parietal areas follows a more intricate dynamic process that is hard to identify with simple correlation analyses (Churchland et al., 2010). If this was the case, an interesting follow-up question would be why the representations of accumulated evidence in parietal and motor, or posterior cingulate areas apparently differ, as we clearly found correlations with accumulated evidence in the latter areas.

We systematically manipulated decision evidence by changing the position of a single dot. Only the x-coordinates of these dot positions represented momentary decision evidence while the decision-irrelevant y-coordinates acted as a perceptual control variable. While this interpretation is dictated by an ideal observer model of our task, in reality it could still be that people also used the y-coordinate for making their decision, for example, by determining the Euclidean distance of the dot to the two beehives. In how far people actually do this is unclear, but we can at least say that people's choices were largely unaffected by the y-coordinate (Figure 3). We therefore believe that the behavioral relevance of the y-coordinate was minimal in our task and that the x-coordinate is a sufficiently good proxy of momentary decision evidence for identifying momentary evidence representations in the brain.

We have shown that correlations of MEG signals with the y-coordinates, in contrast to momentary evidence, were strongly diminished in the period from 300 to 500 ms after dot onset. This suggests that the brain ceases to represent perceptual information that is behaviorally irrelevant around this time and that brain areas with strong correlations with momentary evidence in this time window indeed are involved in the decision making process. This interpretation is further supported by previous work which has shown that purely perceptual stimulus information is represented in electrophysiological signals only until about 400 ms after stimulus onset (Wyart et al., 2012; Mostert et al., 2015; Myers et al., 2015) while specifically decision-related information is represented longer starting around 170 ms after stimulus onset (Philiastides and Sajda, 2006; Philiastides et al., 2006, 2014; Wyart et al., 2012; Mostert et al., 2015; Myers et al., 2015).

We further validated this interpretation by investigating correlations with accumulated evidence, that is, the cumulative sum of momentary evidences within a trial. In contrast to the momentary evidence, this is more specifically related to the decision and has no simple, purely perceptual interpretation. The similarity of the correlation topographies for accumulated evidence and momentary evidence in the late phase suggests that specifically decision-relevant evidence is represented in the late phase, within 300 to 500 ms after evidence updates. Our results do not allow to clearly state whether momentary, or accumulated, or both types of decision evidence were represented in the brain in this time window, because both types of evidence are correlated, especially early within a trial. However, we also found that accumulated evidence exhibited the corresponding central topography more consistently throughout peri-stimulus time than momentary evidence, so it appears reasonable to assume that accumulated evidence is predominantly represented in the late phase.

Finally, and perhaps most surprisingly, we found significant correlations with momentary and accumulated evidence in posterior cingulate cortex across all phases. Especially a ventral part of posterior cingulate cortex (v23ab) was involved already in the early phase which was dominated by early visual areas and may therefore relate to basic visual processing of the stimulus. In the intermediate phase, the correlations were weaker, but persisted. In the late phase it constituted one of the main effects suggesting that it is a region contributing to the maintenance and accumulation of momentary evidence in the brain. Consequently, posterior cingulate cortex appears to be involved in both early sensory processing and decision making and, therefore, could act as a bridge between these processes.

Previous studies investigating the function of posterior cingulate cortex have mostly concentrated on a rather slow time scale, for example, contrasting different task conditions, while we analyzed rapid fluctuations of neural signals. These studies of slow activities in posterior cingulate cortex have implicated its role in directing the focus of attention (Leech and Sharp, 2014). However, posterior cingulate cortex has been associated with a wide range of functions which summarize to estimating the need to change behavior in light of new, external requirements (Pearson et al., 2011). Our findings are compatible with this view, in the context of fast perceptual decision making where participants need to decide whether to follow one (press left) or another (press right) behavior.

In the field of perceptual decision making, especially in electrophysiological work with non-human animals, the posterior cingulate cortex has not gained much attention (Gold and Shadlen, 2007; Hanks and Summerfield, 2017). Therefore, given our findings it appears that the role of posterior cingulate cortex in perceptual decision making may have been underestimated.

## Data Availability Statement

The datasets generated for this study are available on request to the corresponding author.

## Ethics Statement

This study has been approved by the ethics committee of the Technical University of Dresden (EK324082016). Written informed consent was obtained from all participants.

## Author Contributions

SB, HP, KK, and SK contributed conception and design of the study. HP implemented experiment and collected data. SB, HP, and BM preprocessed MEG data. SB performed the statistical analysis and wrote the first draft of the manuscript. All authors contributed to manuscript revision, read, and approved the submitted version.

### Conflict of Interest

The authors declare that the research was conducted in the absence of any commercial or financial relationships that could be construed as a potential conflict of interest.

## Supplementary Material

Supplementary Data Sheet 1 This file contains all significant effects identified from the correlation analysis between regressors of interest and source signals (Main manuscript section ‘Identification of significant source-level effects’).
