# Supplementary material for: Representation of Perceptual Evidence in the Human Brain Assessed by Fast, Within-Trial Dynamic Stimuli
Source: Front Hum Neurosci. 2020 Feb 4;14:9. doi: 10.3389/fnhum.2020.00009 (PMC7010639; doi:10.3389/fnhum.2020.00009)
Supplement: Supplementary Data Sheet 1 — This file contains all significant effects identified from the correlation analysis between regressors of interest and source signals (Main manuscript section ‘Identification of significant source-level effects’). [file Data_Sheet_2.PDF]

Supplementary Material

1 CORRELATIONS BETWEEN MOMENTARY AND ACCUMULATED EVIDENCE

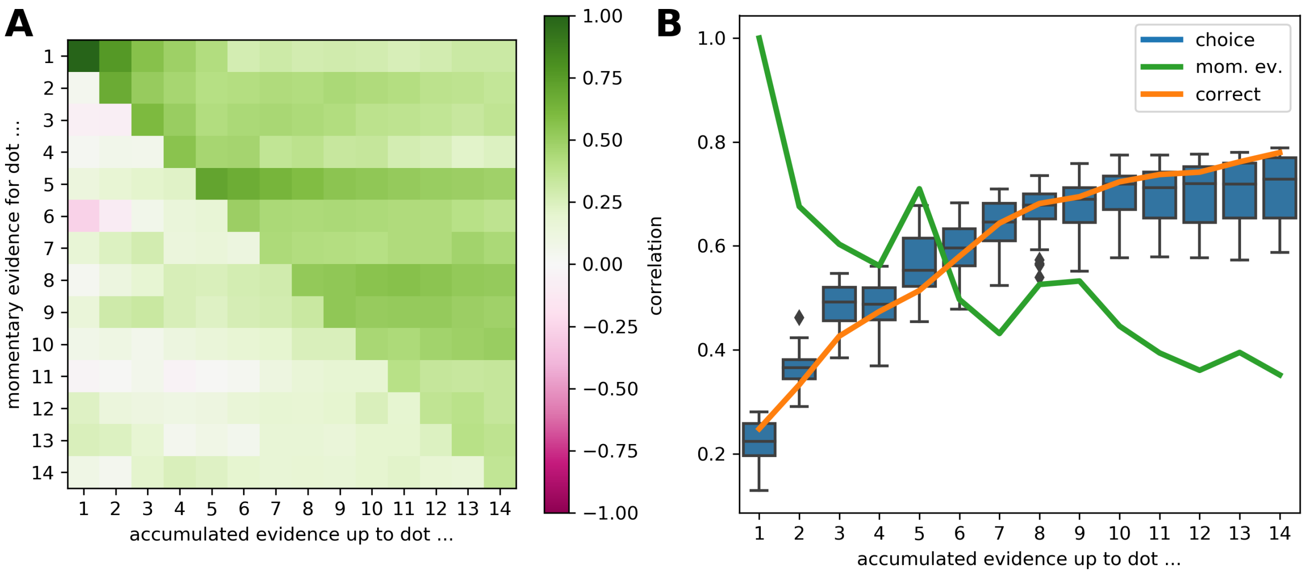

Figure S1. The accumulated evidence is correlated across trials with the momentary evidence provided by dot positions, the correct choice in a trial and the choices of the participants. A: Correlation coefficients for all combinations of momentary and accumulated evidence for the shown onset times. For example, the correlation value at row 2, column 4 gives the correlation between the momentary evidence of the 2nd dot position within a trial and the accumulated evidence up to the 4th dot position, across trials. B: Comparison of correlations between accumulated evidence and three trial-wise measures: the correct choice in a trial (orange line), the momentary evidence at the same time point (green line, equal to diagonal in A), and the choices of the participants (blue boxes). The blue boxes show the distribution over participants per considered dot position.

2 GENERAL LINEAR MODELS

All our regression analyses used general linear models of the form

$$\mathbf{y} = \mathbf{X}\boldsymbol{\beta} + \epsilon \tag{S1}$$

$$= \sum_{i=1}^{R} \beta_i \mathbf{x}_i + \epsilon \tag{S2}$$

where $\mathbf{y}$ are the data to be explained, e.g., magnetometer measurements at a given time point across trials, $\mathbf{X}$ is a design matrix consisting of several regressors, one per column, that could explain the data, $\boldsymbol{\beta}$ are a set of parameters which need to be inferred and determine how well a regressor explains the data, R is the total number of regressors and ϵ is Gaussian noise. So by defining what the data and the regressors are the regression model is fully determined. For clarity we detail below the data and regressor definitions of the regression models described in the main text ordered by results figures.

Note that we normalised data, $\mathbf{y}$, to mean 0 and standard deviation 1 within time points across trials. We also normalised regressors, $\mathbf{x}_i$, to mean 0 and standard deviation 1 except for the intercepts and regressors for which the mean was below 2 * standard deviation. We only scaled these regressors to standard deviation 1 by dividing by their standard deviation without shifting the mean explicitly. This procedure was meant so that positive regressors, such as trial counts, can maintain their conceptual property of only increasing measured values in the model as long as this did not introduce strong collinearity with the intercept. Note that our primary regressors of interest related to the x-coordinates of dots had generally very low mean compared to the standard deviation, because dot x-coordinates were balanced across trials in the experimental design. As a result their mean always was close to 0 after normalisation.

2.1 Figure 4

standard regression analysis

- $\mathbf{y}$ preprocessed magnetometer measurements of sensor MEG2241 for one time point and participant across trials
- $\mathbf{x}_{1-6}$ x-positions of the first 6 dots in these trials
- $\mathbf{x}_{7-12}$ y-positions of the first 6 dots in these trials
- $\mathbf{x}_{13}$ response of the participant in these trials
- $\mathbf{x}_{14}$ trial count, i.e., for each trial an integer telling when in the experiment the trial occurred for this subject
- $\mathbf{x}_{15}$ intercept

2.2 Figure 5

expanded regression analysis

- $\mathbf{y}$ preprocessed magnetometer measurements of one dot onset time, one sensor and participant across times and trials, i.e., given a desired dot onset time several time points in a trial will be included depending on the dot position changes that occurred in that trial (see description in Methods of main text)
- $\mathbf{x}_1$ x-coordinate(s) of the white dot (one for each time point in that trial as included in $\mathbf{y}$ - this is the same for all regressors)
- $\mathbf{x}_2$ y-coordinate(s) of the white dot
- $\mathbf{x}_3$ absolute value of x-coordinate(s)
- $\mathbf{x}_4$ absolute value of y-coordinate(s)
- $\mathbf{x}_5$ perceptual update of x-coordinate(s): $|x_i - x_{i-1}|$
- $\mathbf{x}_6$ perceptual update of y-coordinate(s): $|y_i - y_{i-1}|$
- $\mathbf{x}_7$ accumulated evidence up to previous dot: if the x-coordinate associated with the corresponding data point is that of the i-th shown dot position, then this is accumulated evidence (sum of x-coordinates, but see Methods in main text) up to dot position $i - 1$
- $\mathbf{x}_8$ sum of y-coordinates up to dot position $i - 1$
- $\mathbf{x}_9$ intercept

2.3 Figure 6

expanded regression analysis

y as for Figure 5
x_1 absolute value of x-coordinate(s)
x_2 absolute value of y-coordinate(s)
x_3 perceptual update of x-coordinate(s): $|x_i - x_{i-1}|$
x_4 perceptual update of y-coordinate(s): $|y_i - y_{i-1}|$
x_5 sum of x-coordinates up to dot position i
x_6 sum of y-coordinates up to dot position i
x_7 intercept

2.4 Figure 7

expanded regression analysis

y as for Figure 5, but on reconstructed source currents than on magnetometer measurements
x as for Figure 5

2.5 Figure 8

expanded regression analysis

y as for Figure 6, but on reconstructed source currents than on magnetometer measurements
x as for Figure 6

2.6 Figure 9

response-aligned regression analysis

y preprocessed magnetometer measurements of one sensor, for one response-aligned time point and participant across trials
x_1 choice of participant for the corresponding trial (encoded as -1 and 1)
x_2 trial count, i.e., for each trial an integer telling when in the experiment the trial occurred for this subject
x_3 intercept

2.7 Figure 10

response-aligned regression analysis

y reconstructed source currents of one brain area for one selected time window and participant across response-aligned times and trials, i.e., all response-aligned time points within the selected time window were included for each of the included trials
x as for Figure 9, but expanded to cover the extra data points
